# Molecular Insight into the Association Between Cartilage Regeneration and Ear Wound Healing in Genetic Mouse Models: Targeting New Genes in Regeneration

**DOI:** 10.1534/g3.113.007302

**Published:** 2013-11-01

**Authors:** Muhammad Farooq Rai, Eric J. Schmidt, Audrey McAlinden, James M. Cheverud, Linda J. Sandell

**Affiliations:** * Department of Orthopaedic Surgery, Washington University School of Medicine at Barnes-Jewish Hospital, St. Louis, Missouri 63110; † Cell Biology and Physiology, Washington University School of Medicine at Barnes-Jewish Hospital, St. Louis, Missouri 63110; ‡ Biomedical Engineering, Washington University School of Medicine at Barnes-Jewish Hospital, St. Louis, Missouri 63110; § Anatomy and Neurobiology, Washington University School of Medicine at Barnes-Jewish Hospital, St. Louis, Missouri 63110

**Keywords:** tissue regeneration, articular cartilage, QuantiGene Plex assay, recombinant inbred lines, osteoarthritis

## Abstract

Tissue regeneration is a complex trait with few genetic models available. Mouse strains LG/J and MRL are exceptional healers. Using recombinant inbred strains from a large (LG/J, healer) and small (SM/J, nonhealer) intercross, we have previously shown a positive genetic correlation between ear wound healing, knee cartilage regeneration, and protection from osteoarthritis. We hypothesize that a common set of genes operates in tissue healing and articular cartilage regeneration. Taking advantage of archived histological sections from recombinant inbred strains, we analyzed expression of candidate genes through branched-chain DNA technology directly from tissue lysates. We determined broad-sense heritability of candidates, Pearson correlation of candidates with healing phenotypes, and Ward minimum variance cluster analysis for strains. A bioinformatic assessment of allelic polymorphisms within and near candidate genes was also performed. The expression of several candidates was significantly heritable among strains. Although several genes correlated with both ear wound healing and cartilage healing at a marginal level, the expression of four genes representing DNA repair (*Xrcc2*, *Pcna*) and Wnt signaling (*Axin2*, *Wnt16*) pathways was significantly positively correlated with both phenotypes. Cluster analysis accurately classified healers and nonhealers for seven out of eight strains based on gene expression. Specific sequence differences between LG/J and SM/J were identified as potential causal polymorphisms. Our study suggests a common genetic basis between tissue healing and osteoarthritis susceptibility. Mapping genetic variations causing differences in diverse healing responses in multiple tissues may reveal generic healing processes in pursuit of new therapeutic targets designed to induce or enhance regeneration and, potentially, protection from osteoarthritis.

Regeneration potential is phylogenetically dispersed among animal taxa and is a basal trait in vertebrates, including fishes, amphibians, and mammalian fetal tissues (Gierer *et al.* 1972; Stocum 1984; Fini 1999; Harty *et al.* 2003; Tanaka 2003; Bely 2010; Cuervo *et al.* 2012; Seifert *et al.* 2012). A consensus is that the regenerative capability in adult mammals is extremely limited (ten Koppel *et al.* 2001; Harty *et al.* 2003; Carlson 2005; Colwell *et al.* 2005; Sanchez Alvarado and Tsonis 2006; Kierdorf and Kierdorf 2011). However, at the end of 20th century, the MRL/MpJ mouse emerged as a classical example of mammalian regeneration because it can heal ear wounds (Clark *et al.* 1998; Metcalfe *et al.* 2006; Fitzgerald *et al.* 2008; Rai *et al.* 2012), surgical wounds (Colwell *et al.* 2006; Heydemann *et al.* 2012), and articular cartilage lesions (Fitzgerald *et al.* 2008; Rai *et al.* 2012). In addition, DBA/1 (Kench *et al.* 1999; Li *et al.* 2001; Masinde *et al.* 2006) and LG/J (Kench *et al.* 1999; Li *et al.* 2001; Masinde *et al.* 2006; Rai *et al.* 2012) carry similar regenerative abilities to adulthood. This is in contrast to all other mouse strains tested so far (C57BL/6J, SJL/J, BALB/cByJ, SM/J), in which scar tissue forms at wound sites (Clark *et al.* 1998; Metcalfe *et al.* 2006; Fitzgerald *et al.* 2008; Rai *et al.* 2012).

Failure to regenerate injured articular cartilage poses a great challenge to musculoskeletal research because it is associated with osteoarthritis. Osteoarthritis is a complex heterogeneous disease in which cartilage degenerates because of aging, trauma, genetics or other unknown etiological agents. We have previously shown that an inverse relationship exists between cartilage healing and osteoarthritis susceptibility (Hashimoto *et al.* 2012). The super-healer MRL mouse is protected from posttraumatic osteoarthritis as well (Ward *et al.* 2008). Therefore, study of the genes associated with cartilage regeneration bears great relevance to osteoarthritis research.

A genome-wide microsatellite mapping study has shown that ear wound healing is a complex multigenic trait that is controlled by five to six loci segregating in F_2_ and backcrosses between MRL/MpJ and C57BL/6J (McBrearty *et al.* 1998; Heber-Katz 1999). The LG/J inbred strain has a similar healing ability and is one of the founding lines used to breed the MRL/MpJ strain. Both strains share 75% of their genome (Murphy and Roths 1979). Another mapping study in the F_2_ population derived from LG/J and nonhealing SM/J parental lines detected segregation at four loci that affect ear wound healing, three replicating loci mapped in the MRL/MpJ study plus one novel locus (Blankenhorn *et al.* 2009).

The complex process of regeneration is characterized by spontaneous initiation of several genes and biological pathways (Gurtner *et al.* 2008). Several studies have compared the gene expression in ear wound tissues between healers and nonhealers (McBrearty *et al.* 1998; Li *et al.* 2001; Masinde *et al.* 2006; Blankenhorn *et al.* 2009), generating important information regarding molecular biology of regeneration. The gene expression differences in ear tissues from LGXSM intercross have shown a differential regulation of ∼600 genes within the mapped loci affecting healing (Blankenhorn *et al.* 2009). However, no strong evidence is yet available for the genes that control cartilage regeneration in these strains.

We have recently studied ear wound healing and cartilage regeneration simultaneously in a set of recombinant inbred (RI) lines generated from LG/J (healer) and SM/J (nonhealer) intercross (Rai *et al.* 2012). We found a strong correlation between the two phenotypes, indicating a common genetic basis of healing and indicating that the underlying mechanisms might not be tissue-specific. A recent study has shown that distinct differences in cell-cycle properties exist between healer and nonhealer strains (Bedelbaeva *et al.* 2010). These data suggest that highly regenerative cells are both hyperproliferative and highly apoptotic in nature. The combined effects of increased proliferation and apoptosis might allow the organism to eliminate old cells and keep the cell turnover rate high, as seen in some organ development. However, the molecular and cellular basis for differences in regeneration is not completely understood.

In this study, we harvested injured knee joint tissues from formalin-fixed paraffin-embedded (FFPE) sections archived from common inbred and RI strains and measured the expression signatures of mRNAs and microRNAs (miRNAs) using a novel QuantiGene Plex assay directly from tissue lysates. We hypothesize that the healing responses have a common genetic basis, implying a common suite of mechanisms. We believe that identification of biological processes common to regeneration/healing in articular cartilage has novel implications for osteoarthritis pathogenesis and therapeutic interventions.

## Materials and Methods

### Mice source and housing

All procedures in this study were approved by the Animal Studies Committee of Washington University. Eight mouse strains (three to four mice per strain) were used based on previous screening (Rai *et al.* 2012), representing the following three healing categories: good (MRL/MpJ, LG/J, LGXSM-6); intermediate (LGXSM-5, LGXSM-35); and poor (C57BL/6J, SM/J, LGXSM-33). Detailed information and history of RI lines are documented elsewhere (Hrbek *et al.* 2006).

### Phenotyping: ear pinna wound

A 2-mm bilateral hole was produced in the cartilaginous part of the external ear in 6-wk-old mice (McBrearty *et al.* 1998; Rai *et al.* 2012). The hole diameter was read 30 d after the punch and the healing area was calculated by subtracting the diameter of the residual hole from the original 2-mm hole. The phenotypic data for ear wound scores are provided in Supporting Information, Table S1.

### Phenotyping: articular cartilage defect

Full-thickness articular cartilage defect was created through microsurgery on the trochlear groove of 8-wk-old mice (Fitzgerald *et al.* 2008; Rai *et al.* 2012). Harvested knee joints were processed for histology 16 wk postsurgery. FFPE samples were mounted in paraffin blocks, sagittally sectioned at 5-μm intervals, mounted on polylysine-coated slides (Fischer Scientific, Pittsburg, PA), and stained with toluidine blue. The degree of cartilage regeneration was assessed as described previously (Fitzgerald *et al.* 2008; Rai *et al.* 2012). The phenotypic data for articular cartilage regeneration scores are provided in Table S2 (only data from the 16-wk postsurgery time point are related to this study).

### Archived histological sections

The archived unstained FFPE sections (25 sections per strain, three to four mice per strain) from articular cartilage were used to prepare tissue homogenates (“lyse ‘n’ go”) instead of isolating RNA. Previous reports have suggested that the quality of RNA extracted from FFPE tissues is inferior compared to fresh or frozen tissues or cells. Several factors contribute to this compromised quality of RNA. These include formalin fixation (Bresters *et al.* 1994; Macabeo-Ong *et al.* 2002), RNA cross-linking with proteins (Finke *et al.* 1993; Masuda *et al.* 1999), and addition of mono-methyl on all four bases (AUGC) (Park *et al.* 1996; Masuda *et al.* 1999). To overcome these problems and to make use of our valuable archival FFPE tissues, the Affymetrix QuantiGene Plex assay (Panomics Inc., Fremont, CA) was used. This method is based on a sandwich nucleic acid hybridization assay (branch DNA technology) and provides a novel approach for gene expression analysis and quantification (Yang *et al.* 2006) by analyzing the reporter genes rather than target sequences (Urdea *et al.* 1991). There are several advantages associated with the measurement of gene signals directly from FFPE tissue homogenates, including avoidance of variations or errors inherent to RNA extraction and amplification of target sequences. In addition, the sensitivity of branched DNA assay in tissue homogenates has been reported to be 10-fold higher than that of purified RNA (Knudsen *et al.* 2008).

### Preparation of FFPE tissue homogenates

**Figure 1:**
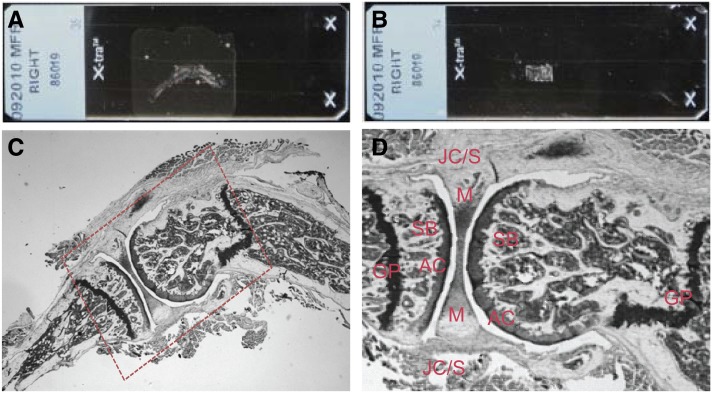
Histological landmarks for tissue collection. The archived FFPE sections were selected from each strain, the surface around the sections was treated with RNaseZAP, and the tibiofemoral joint was visually selected (A, C; dotted area). The excess paraffin was removed around the joint tissues (B, D) and the remaining tissues of interest were collected from 25 sections in a microfuge tube. GP, growth plate; SB, subchondral bone; AC, articular cartilage; M, meniscus; JC/S, joint capsule/synovium.

The area of interest was selectively macrodissected from FFPE sections to collect the composite tissues (subchondral bone, cartilage, meniscus, growth plate, joint capsule, and synovium) (Figure 1). Tissue homogenates were prepared according to the procedure described in Affymetrix QuantiGene Plex sample processing kit for FFPE tissues (Panomics Inc.). All the surfaces and instruments were treated with RNaseZAP (Sigma Aldrich, St. Louis, MO). The excess paraffin was scraped off, along with any tissues around the area of interest. The area of interest on the 5-μm slide section was selectively macrodissected using a clean razor blade and transferred to a 1.5-ml microfuge tube. These crude tissues were incubated at 65° for 6 hr with 600 μl homogenizing solution and 6 μl proteinase K (50 μg/ml). To achieve complete dissociation of the tissues, samples were vortexed several times at 60-min intervals at maximum speed during incubation. The tissue homogenate was separated from debris and paraffin by centrifugation at 14,000 rpm for 5 min at room temperature. The residual paraffin appeared as a solid residuum above the homogenate and debris pelleted at the bottom of the tube. The solid paraffin layer was pierced with a pipette tip to transfer the clear homogenate to a fresh microfuge tube, avoiding contamination with residual paraffin or tissue debris. The resulting homogenate was stored at −80° until being used for QuantiGene Plex and QuantiGene miRNA assays.

### Selection of candidate genes and miRNAs

Sixty-seven mRNAs (Table S3) and six miRNAs (Table S4) were selected for analysis. Individual bead-based oligonucleotide probe sets specific for each mouse target gene were developed by Panomics Inc. using previously published NCBI (National Center for Biotechnology Information) gene accession numbers. The selection of these genes was based on their known role in tissue healing, cartilage homeostasis, joint development, and osteoarthritis. Three reference genes (*Gapdh*, *Hprt1*, and *Actb*) were used to normalize target gene expression. Two panels of genes were submitted for QuantiGene Plex assay, panel 321347 and panel 321389 (http://www.panomics.com).

### Quantification of mRNA expression

The quantification of selected mRNAs in tissue lysates was performed in biological and technical replicates using the Affymetrix QuantiGene 2.0 Plex Assay kit (Panomics Inc.). The assays were performed as described previously with the exception that previously we used RNA instead of tissue lysates (Rai *et al.* 2013). An appropriate volume of working bead mix was prepared by combining the following reagents per well in a microfuge tube and scaled for 96-well plate format: 18.5 μl nuclease-free water; 33.3 μl lysis mixture; 2 μl blocking reagent; 0.2 μl proteinase K; 1 μl capture beads; and 5 μl probe set. The working bead mix was vortexed for 10 sec and 60 μl of it was dispensed into each well of the hybridization plate, followed by addition of 40 μl of tissue homogenate to each well. The plate was sealed using a pressure seal and placed on an inverted plate lid placed onto the VorTemp 56 shaking incubator and incubated for 18−22 hr at 54° while shaking at 600 rpm. Postincubation, the contents of the hybridization plate were transferred to a magnetic separation plate after centrifugation at 240*g* for 60 sec and mixing the contents a few times with a pipette. The magnetic separation plate was inserted into the hand-held magnetic plate washer and the plate was washed three times using 100 μl wash buffer (wash buffer components 1 and 2 in nuclease-free water) per well. Then, 100 μl preamplifier working reagent (36 μl 2.0 preamplifier in 12 ml amplifier diluent) was dispensed into each well of the magnetic separation plate, sealed, and incubated at 50° for 1 hr while shaking at 600 rpm. After incubation, the plate was washed as described, 100 μl amplifier working reagent (36 μL 2.0 amplifier in 12 ml amplifier diluent) was added per well, and incubation was again performed at 50° for 60 min at 600 rpm. Then, the plate was washed again, 100 μL label probe working reagent (36 μl label probe in 12 ml label probe diluent) was added to each well, and the sealed plate was placed into VorTemp and incubated at 50° for 60 min while shaking at 600 rpm. After 60 min, the washing procedure was repeated as described and 100 μl SAPE (Streptavidin conjugated Phycoerythrin) working reagent (36 μl SAPE to 12 ml SAPE diluent) was pipetted into each well. The plate was foiled, removed from the plate washer, and placed on a shaking platform at room temperature while shaking at 800 rpm for 60 sec, followed by shaking at 600 rpm for 30 min. The plate was washed with 130 μl SAPE wash buffer in each assay well and the plate was sealed, removed from the plate washer, wrapped in aluminum foil, and allowed to shake at 800 rpm for 3 min at room temperature. Finally, the plate was read on Luminex set for specified bead regions with the following parameters: 100-μl sample size; 5000–25,000 DD gate; 45-sec timeout; and 100 bead events per bead region. The average signal values were noted, subtracted from the background signal, and finally normalized to housekeeping genes.

### Quantification of miRNA expression

Selected miRNAs were quantified using QuantiGene 2.0 miRNA assay kit (Panomics Inc.) as detailed. Working probe sets for each reaction were prepared by combining the following reagents in a microfuge tube: 25.1 μl nuclease-free water; 33.3 μl lysis mixture; 1 μl blocking reagent; 0.3 μl capture extender; and 0.3 μl label extender. The reaction mixture (60 μl/well) was pipetted into each assay well of the capture plate with conjugated oligonucleotides to the surface of the well. Then, 40 μl sample was dispensed to each assay well, avoiding introduction of bubbles, and 40 μl homogenizing solution was added to the assay background controls. The plate was sealed with adhesive seal and centrifuged at 240*g* for 20 sec at room temperature to ensure the contents would contact the bottom of the well. Finally, the plate was incubated at 46° for 16–20 hr for hybridization. The next day, the plate was first washed three times using 200–300 μl/well of wash buffer (wash components 1 and 2 in distilled water), and then the inverted plate was centrifuged at 240*g* for 60 sec to remove traces of wash buffer. A total of 100 μl/well of freshly prepared PreAmp working reagent (11 μl 2.0 PreAmp in 11 ml amplifier/label diluent) were added to each well of the plate. The plate was sealed and set for PreAmp hybridization at 46° for 60 min. After the incubation period, the plate was washed and centrifuged as described. To each well of the plate, 100 μl Amp working reagent (11 μl 2.0 Amp in 11 ml amplifier/label probe) was added, sealed, and again incubated at 46° for 60 min for Amp hybridization. After the incubation period and washing steps, 100 μl/well label probe was dispensed and kept for hybridization at 46° for 60 min. Finally, the plate was washed after incubation and 100 μl substrate was added to each well of the capture plate, which was sealed and incubated at room temperature for 5 min. The plate was read on a luminometer after removing the seal within 15 min. Average signal from the three replicates was calculated and average background signals were deducted from samples, and the data were normalized to *Snord68*.

### Statistical analyses

All statistical analyses were performed using Systat-12 software (Systat Software Inc., Chicago, IL).

#### Gene expression measurements:

The raw values of mRNA expression levels were normalized using *Gapdh*, *Hprt1*, and *Actb*, whereas those of miRNAs were normalized with *Snord68* as the independent variables in a multiple regression for each of the target genes. The residuals from this regression were obtained for each gene and were used to calculate an average for each individual. The more conventional ΔΔC_t_ method measures differential expression of samples relative to a single reference sample and then compares candidate genes to control genes. However, restating measurements of candidate genes relative to control genes as ratios results in amplification of measurement errors and introduces an artifactual correlation between the ratio plotted on the Y-axis and its denominator plotted on the X-axis.

#### Correlation of gene expression with phenotypic traits:

Pearson correlation was used for examining the associations between gene expression (mRNA or miRNA) values with phenotypic (articular cartilage regeneration or ear wound healing) scores.

#### Heritability of gene expression:

We used ANOVA to test for significant strain differences in gene expression:

$$Y_{ij}=\mu +Sex_{i}+Strain_{j}+e_{ij}$$

with “Sex” as a fixed effect and “Strain” as a random effect. Sex was removed from the model because there were no significant sex differences. Post hoc pairwise significance tests comparing specific mouse strain pairs were performed using Tukey's honestly significant difference test to control for multiple comparisons.

Because these are fully inbred mouse strains, the broad-sense heritability (H^2^) of the traits was calculated using the following equation:

$$H_{trait}^{2}=\sigma_{st}^{2}/\left(\sigma_{st}^{2}+\sigma_{r}^{2}\right)$$

where the variance among strains $\left(\sigma_{st}^{2}\right)$ is divided by the sum of the between-strain $\left(\sigma_{st}^{2}\right)$ and within-strain $\left(\sigma_{r}^{2}\right)$ variances. This includes all sources of genetic variation between mouse strains. Standard errors for heritabilities were calculated using the intraclass correlation (Falconer and Mackay 1996) and were used to generate one-sided 95% confidence intervals (Table 1).

**Table 1:** Heritability and correlations of mRNAs with ear and articular cartilage healing phenotypes

Gene Symbol	Heritability						Correlation with Ear Wound		Correlation with Articular Cartilage		Gene Symbol	Heritability						Correlation with Ear Wound		Correlation with Articular Cartilage	
	Vb	Vw	Vt	H^2^	95% C.I. (One-Tailed)	*P*	*r*	*P*	*R*	*P*		Vb	Vw	Vt	H^2^	95% C.I. (One-Tailed)	*P*	*r*	*P*	*r*	*P*
*Acan*	0.013	0.046	0.059	0.219	0.598	0.126	0.414	0.175	0.488	0.139	*Lef1*	0.000	0.009	0.009	−0.003	0.325	0.466	0.185	0.333	0.375	0.197
*Adamts4*	0.001	0.007	0.008	0.099	0.457	0.282	0.018	0.483	−0.169	0.346	*Lep*	0.000	0.010	0.010	−0.009	0.325	0.481	−0.661	0.078	−0.694	0.070
*Adamts5*	0.003	0.007	0.010	0.279	0.661	0.078	0.163	0.352	0.110	0.398	*Map1lc3a*	0.001	0.003	0.004	0.251	0.632	0.082	−0.770	**0.054**	−0.636	0.085
*Adipoq*	0.012	0.021	0.033	0.375	0.750	**0.022**	−0.530	0.121	−0.714	0.066	*Mia1*	0.016	0.012	0.028	0.575	0.892	**0.002**	0.407	0.179	0.492	0.137
*Apln*	−0.001	0.006	0.005	−0.118	0.325	0.725	0.148	0.364	0.155	0.359	*Mmp13*	0.013	0.017	0.030	0.446	0.964	**0.013**	0.570	0.106	0.562	0.109
*Atg7*	0.016	0.003	0.020	0.824	0.987	**<0.001**	−0.223	0.302	−0.196	0.324	*Mmp1a*	0.000	0.007	0.006	−0.013	0.807	0.489	−0.371	0.199	−0.353	0.210
*Axin2*	0.003	0.008	0.011	0.258	0.639	0.077	0.802	**0.049**	0.814	**0.047**	*Mmp3*	0.028	0.091	0.119	0.234	0.325	0.113	0.444	0.159	0.294	0.249
*Bcl3*	0.007	0.011	0.018	0.377	0.751	**0.030**	−0.353	0.210	−0.566	0.107	*Mmp9*	0.020	0.007	0.027	0.737	0.614	**<0.001**	−0.157	0.357	−0.039	0.463
*Becn1*	0.002	0.004	0.006	0.356	0.733	**0.028**	−0.127	0.383	−0.033	0.469	*Nampt*	0.000	0.002	0.002	−0.114	0.325	0.716	0.335	0.222	0.108	0.400
*Bglap1*	0.027	0.016	0.043	0.636	0.924	**<0.001**	−0.127	0.383	−0.033	0.469	*Nfkb1*	0.001	0.002	0.002	0.256	0.637	0.094	−0.446	0.158	−0.535	0.119
*Bmp6*	0.000	0.003	0.003	−0.133	0.325	0.758	−0.195	0.325	−0.114	0.395	*Pcna*	0.002	0.007	0.009	0.209	0.587	0.119	0.893	**0.036**	0.925	**0.032**
*Casp3*	0.003	0.004	0.008	0.441	0.803	**0.014**	0.712	0.066	0.740	0.060	*Polq*	0.008	0.008	0.016	0.477	0.830	**0.005**	0.104	0.404	0.126	0.384
*Ccl3*	0.003	0.065	0.068	0.044	0.386	0.378	−0.335	0.222	−0.285	0.256	*Pthlh*	−0.001	0.013	0.012	−0.077	0.325	0.634	0.250	0.281	0.424	0.170
*Cdkn1a*	−0.004	0.016	0.012	−0.318	0.325	0.991	−0.294	0.250	−0.173	0.343	*Retn*	0.000	0.004	0.003	−0.144	0.325	0.779	−0.325	0.228	−0.450	0.156
*Cdkn2a*	0.000	0.009	0.009	0.019	0.352	0.419	0.398	0.184	0.383	0.192	*Runx2*	0.003	0.004	0.007	0.412	0.780	**0.020**	0.654	0.080	0.714	0.066
*Cebpb*	0.002	0.015	0.017	0.138	0.505	0.222	−0.673	0.075	−0.786	**0.051**	*Smad1*	0.002	0.006	0.008	0.217	0.596	0.111	0.411	0.177	0.416	0.174
*Chrdl1*	0.000	0.006	0.007	0.068	0.417	0.321	0.417	0.173	0.385	0.191	*Smad2*	0.000	0.002	0.002	0.131	0.497	0.215	0.155	0.359	0.120	0.389
*Col10a1*	0.004	0.080	0.084	0.043	0.384	0.380	0.014	0.487	0.218	0.306	*Smad4*	0.000	0.002	0.002	0.095	0.452	0.272	0.375	0.197	0.449	0.157
*Col1a1*	0.000	0.009	0.009	−0.044	0.325	0.553	−0.342	0.217	−0.208	0.314	*Sox5*	0.001	0.013	0.014	0.058	0.404	0.339	0.635	0.085	0.683	0.073
*Col2a1*	0.019	0.044	0.062	0.299	0.680	0.065	0.358	0.207	0.417	0.173	*Sox9*	−0.001	0.027	0.026	−0.036	0.325	0.536	0.204	0.318	0.271	0.266
*Col6a6*	−0.001	0.020	0.018	−0.067	0.325	0.601	0.380	0.194	0.269	0.267	*Stat3*	0.000	0.003	0.003	0.129	0.494	0.235	0.044	0.459	−0.152	0.361
*Comp*	0.004	0.042	0.045	0.078	0.430	0.317	0.255	0.278	0.317	0.234	*Stat6*	0.000	0.004	0.004	0.075	0.427	0.323	0.362	0.204	0.389	0.189
*Csnk2a1*	0.000	0.002	0.002	0.046	0.388	0.364	−0.586	0.101	−0.473	0.145	*Tfap2a*	0.002	0.007	0.010	0.250	0.631	0.099	0.112	0.396	0.075	0.431
*Cxcl12*	0.000	0.003	0.004	0.112	0.474	0.261	0.574	0.105	0.580	0.103	*Tgfb1*	0.002	0.003	0.005	0.437	0.800	**0.015**	−0.416	0.174	−0.466	0.148
*Ddr2*	0.005	0.012	0.017	0.293	0.674	0.068	0.124	0.386	0.157	0.357	*Tgfbr1*	0.001	0.003	0.003	0.209	0.587	0.137	−0.199	0.322	−0.196	0.324
*Epas1*	0.001	0.002	0.004	0.375	0.750	**0.031**	−0.067	0.438	−0.063	0.441	*Trp53*	0.000	0.003	0.003	0.123	0.487	0.226	−0.103	0.404	−0.338	0.220
*Fancc*	0.000	0.003	0.003	−0.036	0.325	0.541	0.641	0.084	0.689	0.071	*Ulk1*	0.003	0.003	0.006	0.475	0.828	**0.006**	0.643	0.083	0.773	**0.054**
*Fgf18*	0.015	0.023	0.039	0.396	0.767	**0.024**	−0.196	0.324	−0.265	0.270	*Vegfa*	0.001	0.006	0.007	0.132	0.498	0.214	−0.141	0.370	−0.128	0.383
*Fzd2*	0.000	0.005	0.004	−0.101	0.325	0.687	0.561	0.109	0.643	0.083	*Wisp1*	0.008	0.011	0.019	0.433	0.797	**0.010**	0.320	0.232	0.356	0.208
*Gdf5*	0.008	0.024	0.032	0.247	0.628	0.102	−0.112	0.397	−0.198	0.322	*Wnt16*	−0.002	0.019	0.016	−0.138	0.325	0.743	0.776	**0.053**	0.868	**0.039**
*Igf1*	0.017	0.017	0.034	0.505	0.849	**0.006**	−0.184	0.334	−0.215	0.308	*Wnt3a*	0.000	0.008	0.008	0.059	0.406	0.349	0.052	0.451	−0.196	0.324
*Ihh*	0.011	0.010	0.022	0.529	0.864	**0.002**	0.325	0.228	0.448	0.157	*Xpa*	−0.001	0.004	0.003	−0.171	0.325	0.832	0.714	0.065	0.526	0.123
*Il4*	0.002	0.005	0.007	0.247	0.628	0.102	0.090	0.416	−0.085	0.421	*Xrcc2*	0.006	0.007	0.013	0.493	0.841	**0.004**	0.894	**0.035**	0.818	**0.046**
*Il6*	−0.001	0.009	0.008	−0.087	0.325	0.643	0.820	**0.046**	0.755	0.057											

Vb, between-strain variation; Vw, within-strain variation; Vt, total phenotypic variation; H^2^ = heritability; C.I., confidence interval. Bold values indicate significance at the 5% level.

### Single nucleotide polymorphisms analysis

Utilizing whole-genome sequences for LG/J at 21-times coverage and SM/J at 14-times coverage (H. A. Lawson, I. Nikolskiy, S. Chun, M. McLellan, J. Fay, E. Mardis, J. M. Cheverud, unpublished data), we evaluated candidate genes for single nucleotide polymorphisms (SNPs). For each gene, SNPs were identified in the 5′ and 3′ UTRs, exons, introns, and the regions 2500 base pairs upstream and downstream of the UTRs. The sequences of genic elements were according to the UCSC genome browser annotations, NCBI37/mm9 assembly (http://genome.ucsc.edu/). Conservation scores for each SNP were obtained from the PhastCons30Placental table of the UCSC browser. Nonsynonymous SNPs with a conservation score of 0.90 or greater were evaluated for potential functional significance using the algorithms PolyPhen-2 (Adzhubei *et al.* 2010, 2013), SIFT (Kumar *et al.* 2009), and LRT (Chun and Fay 2009). PolyPhen-2 uses sequence-based predictive features in light of tertiary structures of proteins to predict the functional significance of nonsynonymous amino acid substitutions. SIFT makes use of sequence homology and median evolutionary conservation scores to predict conserved protein function. LRT is a likelihood ratio test comparing the probability that a codon has evolved under negative selection to a model in which the codon has evolved neutrally, where rates of synonymous and nonsynonymous substitutions are equal. Genetic maps with the locations of SNPs and microsatellite markers are available for the RI lines (Hrbek *et al.* 2006). For each gene, the genotype in each RI line was inferred by finding the genotypes of the closest set of flanking markers in the appropriate RI line genetic map. When a gene was between proximal and distal RI map markers that differ in genotype, a genotype was not assigned.

### Fold-change differences in gene expression and cluster analysis

We used *t* test and probabilities of obtaining the observed results given the null hypothesis of less than 5% is accepted as statistically significant. The fold-changes in gene expression were calculated using the least square means for each strain against C57BL/6J and heat maps were generated to visualize the magnitude of fold-change variations. We used Ward minimum variance method (cluster analysis) to determine strain inter-relationships of gene expression correlation strengths.

## Results

### Findings of the broad-sense heritabilities

H^2^ showed that the expression levels of 19 mRNAs (Table 1) and six miRNAs (Table 2) were significantly heritable, indicating that the variation in gene expression corresponded to genetic differences among strains. The heritabilities of mRNAs ranged from 35.6% to 82.4%, whereas those of miRNAs ranged from 60.0% to 83.9%. There were six genes (*Igf1*, *Ihh*, *Mia1*, *Bglap*, *Mmp9*, and *Atg7*) that showed a statistically significant heritability of 50% or more. The highest heritability was found to be for *Atg7* (82.4%; *P* < 0.001), followed by *Mmp9* (73.3%; *P* < 0.001) and *Bglap1* (63.6%; *P* < 0.001). For miRNAs, the heritability was more than 50%, with miR-17 being the most significantly heritable (H^2^ = 83.9%; *P* < 0.001). Among the rest, miR-224 (H^2^ = 81.8%; *P* < 0.001), miR-146b–5p (H^2^ = 80.4%; *P* < 0.001), miR-27b–3p (H^2^ = 64.0%; *P* = 0.001), miR-675–5p (H^2^ = 62.5%; *P* = 0.001), and miR-140–5p (H^2^ = 60.0%; *P* = 0.002) were also significantly heritable.

**Table 2:** Heritability and correlations of miRNAs with ear and articular cartilage healing phenotypes

miRNA	Heritability						Correlation with Ear Wound		Correlation with Articular Cartilage	
	Vb	Vw	Vt	H^2^	95% C.I. (One-Tailed)	*P*	*r*	*P*	*r*	*P*
miR-17	0.41	0.08	0.49	0.84	0.990	**<0.001**	0.083	0.422	−0.128	0.382
miR-27b-3p	0.49	0.28	0.77	0.64	0.926	**0.001**	0.258	0.276	−0.288	0.253
miR-140-5p	0.62	0.41	1.03	0.6	0.906	**0.002**	0.016	0.485	−0.169	0.347
miR-146b-5p	0.37	0.09	0.45	0.8	0.983	**<0.001**	0.197	0.324	−0.148	0.364
miR-224	0.41	0.09	0.5	0.82	0.986	**<0.001**	−0.013	0.487	−0.147	0.366
miR-675	0.3	0.18	0.48	0.63	0.918	**0.001**	0.601	0.096	−0.580	0.102

miRNA, microRNA; Vb, between-strain variation; Vw, within-strain variation; Vt, total phenotypic variation; H^2^, heritability; C.I., confidence interval. Bold values indicate significance at the 5% level.

### Findings of the association between gene expression signatures and healing scores

**Figure 2:**
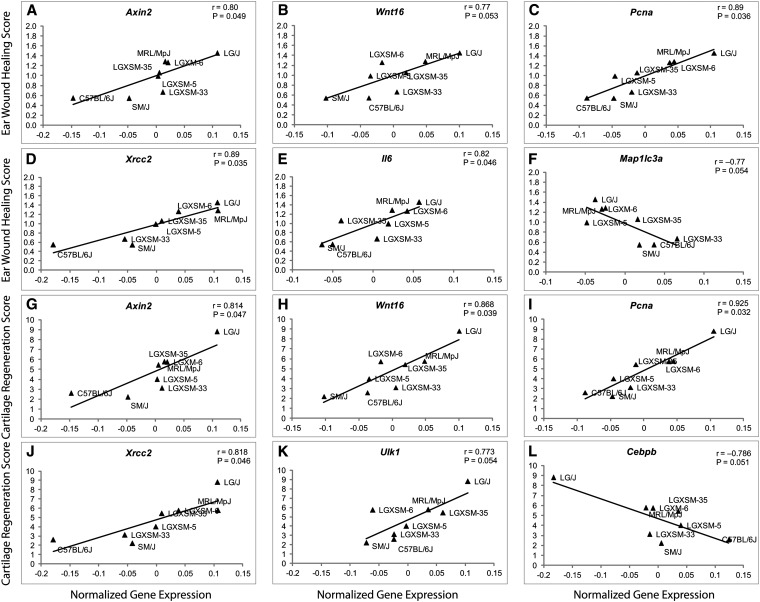
Genes significantly correlated with ear and articular cartilage healing phenotypes. Knee joint tissues from FFPE sections were macrodissected and tissue homogenates were prepared. The gene expression levels were quantified through QuantiGene Plex assay and were correlated with ear and articular cartilage healing scores. Five genes, namely *Axin2*, *Wnt16*, *Pcna*, *Xrcc2*, and *Il6*, were found to be significantly positively correlated with ear wound healing, whereas *Map1lc3a* was significantly negatively correlated with ear wound healing (A–F). Similarly, five genes, namely *Axin2*, *Wnt16*, *Pcna*, *Xrcc2*, and *Ulk1*, were found to be significantly positively correlated, whereas *Cebpb* was significantly negatively correlated with articular cartilage healing (G–L). *r* = Pearson correlation coefficient.

Ear wound and cartilage healing scores (Y-axis of respective graphs) were plotted against gene expression levels (X-axis of each graph) (Figure 2) to determine significance of correlation. Six gene expression traits were significantly correlated with both ear wound and articular cartilage phenotypes, indicating that regulation of these genes plausibly orchestrates tissue healing. The genes that were strongly correlated with ear wound healing were *Xrcc2* (*r* = 0.894; *P* = 0.035), *Pcna* (*r* =0.893; *P* = 0.036), *Axin2* (*r* = 0.802; *P* = 0.049), *Wnt16* (*r* = 0.776; *P* = 0.053), *Il6* (*r* = 0.820; *P* = 0.046), and *Map1lc3a* (*r* = −0.770; *P* = 0.054). The genes that were most strongly associated with articular cartilage regeneration were *Pcna* (*r* = 0.925; *P* = 0.032), followed by *Wnt16* (*r* = 0.868; *P* = 0.039), *Xrcc2* (*r* = 0.818; *P* = 0.046), *Axin2* (*r* = 0.814; *P* = 0.047), *Cebpb* (*r* = −0.786; *P* = 0.051), and *Ulk*1 (*r* = 0.773; *P* = 0.054). Interestingly, four of these genes were common to both ear wound healing and articular cartilage regeneration (*Xrcc2*, *Pcna*, *Axin2*, and *Wnt16)*. *Il6* and *Map1lc3a* correlated with ear wound healing and correlated in the same direction with articular cartilage regeneration with borderline significance (*P* = 0.057 and *P* = 0.085, respectively). Likewise, the *Cebpb* and *Ulk1*, which were significantly correlated with articular cartilage regeneration, were also correlated with ear wound healing with borderline significance (*P* = 0.083 and *P* = 0.075, respectively). In addition, there were several other genes that were correlated with both phenotypes at a probability of 10%, including *Adipoq*, *Casp3*, *Mmp13*, *Runx2*, *Csnk2a1*, *Cxcl12*, *Fancc*, *Fzd2*, and *Sox5*. The association of gene expression with each phenotype and levels of significance are presented in Table 1. None of the miRNAs was significantly associated with healing (Table 2).

Notably, of the majority of genes most strongly correlated with both phenotypes, the heritability estimates are nonsignificant (Table 1). However, given the relatively small number of strains assessed, the standard error for an estimate of 0% heritability approaches 30%, so that heritability estimates must be greater than 50% to be significant.

### Findings of the inter-relation of strains based on gene expression signatures

**Figure 3:**
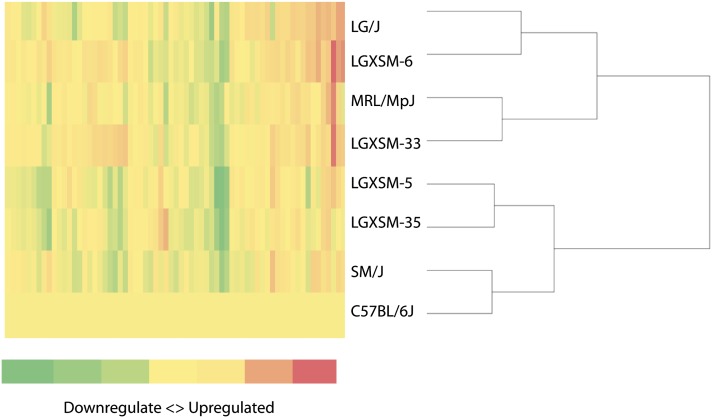
Cluster analysis of normalized gene expression levels. Knee joint tissues from FFPE sections were macrodissected and tissue homogenates were prepared to quantify candidate genes through QuantiGene Plex assay. The gene expression levels were used to calculate fold difference among strains compared to C57BL/6J (a nonhealer control strain) as shown in a seven-tiered categorical scale. Hierarchical clustering analysis was performed using Ward minimum variance method to produce a dendrogram. As shown here, the healer strains (except for LGXSM-33) clustered together exclusive of other nonhealers and intermediate healers, indicating an association between gene expression levels and healing potential.

The most interesting finding from the cluster analysis was the classification of strains into two distinct clusters largely (but not exclusively) corresponding to their healing ability (Rai *et al.* 2012) with 100% bootstrap support (1000 iterations) (Figure 3). One cluster represented nonhealers and moderate healers, including SM/J, C57BL/6J, LGXSM-5, and LGXSM-35, whereas the other cluster represented predominately classical healers LG/J, LGXSM-6, and MRL/MpJ. Surprisingly, strain LGXSM-33, a poor healer, was clustered with MRL/MpJ, a super-healer.

### Findings of the SNPs analysis

We assessed composition and distribution of SNPs in the genes showing correlation at a significant (*P* < 0.05) or marginally significant (*P* < 0.1) level (Table 3). We found a few SNPs occupying evolutionary conserved positions between alleles of LG/J and SM/J parental lines as discussed.

**Table 3:** SNPs in genes correlated with ear and articular cartilage healing phenotypes

Gene Symbol	Chromosome	Genotype				Total SNPs	Highly Conserved Site	Total Noncoding	Highly Conserved Site	Noncoding DNA										Coding DNA							
		LGXSM-6	LGXSM-35	LGXSM-5	LGXSM-33					Upstream 2500 bp	Highly Conserved Site	5′ UTR	Highly Conserved Site	Intronic SNPs	Highly Conserved Site	3′ UTR	Highly Conserved Site	Downstream 2500 bp	Highly Conserved Site	Exonic SNPs	Highly Conserved Site	Synonymous	Nonsynonymous	Significance*^a^*	Position	Exon	Substitution
*Axin2*	11	SS	SS	LL	SS	37	4	32	1	1	0	0	0	30	1	1	0	0	0	5	3	3	2	Np; PolyPhen-2	108,805,010; 108,803,724	5; 7	Arg:648:Cys; Tyr:474:His
*Wnt16*	6	LL	LL	?*^b^*	SS	81	5	79	5	5	0	3	1	47	3	3	0	21	1	2	0	2	0	Np	—	—	—
*Pcna*	2	LL	SS	?	SS	6	1	5	0	2	0	0	0	1	0	0	0	2	0	1	1	1	0	Np	—	—	—
*Xrcc2*	5	LL	LL	LL	SS	100	4	98	2	30	0	0	0	59	2	2	0	7	0	2	2	1	1	Np	25,204,113	2	Gly:26:Ala
*Il6*	5	LL	LL	LL	SS	0	0	0	0	0	0	0	0	0	0	0	0	0	0	0	0	0	0	—	—	—	—
*Cebpb*	2	?	LL	LL	SS	0	0	0	0	0	0	0	0	—*^c^*	—	0	0	0	0	0	0	0	0	—	—	—	—
*Map1lc3a*	2	LL	LL	LL	?	32	1	32	1	16	0	0	0	2	0	0	0	14	1	0	0	0	0	—	—	—	—
*Ulk1*	5	LL	SS	SS	LL	4	0	3	0	2	0	0	0	1	0	0	0	0	0	1	0	1	0	Np	—	—	—
*Adipoq*	16	LL	SS	LL	?	23	0	23	0	4	0	0	0	12	0	2	0	5	0	0	0	0	0	—	—	—	—
*Casp3*	8	LL	?	LL	LL	59	0	59	0	0	0	0	0	58	0	1	0	0	0	0	0	0	0	—	—	—	—
*Csnk2a1*	2	LL	?	LL	SS	1	0	1	0	0	0	0	0	1	0	0	0	0	0	0	0	0	0	—	—	—	—
*Cxcl12*	6	LL	SS	LL	SS	0	0	0	0	0	0	0	0	0	0	0	0	0	0	0	0	0	0	—	—	—	—
*Fancc*	13	SS	LL	LL	LS	23	0	23	0	1	0	0	0	22	0	0	0	0	0	0	0	0	0	—	—	—	—
*Fzd2*	11	SS	LL	?	SS	13	2	13	2	5	1	1	1	–*^c^*	—	1	0	6	0	0	0	0	0	—	—	—	—
*Mmp13*	9	LL	SS	LL	LL	0	0	0	0	0	0	0	0	0	0	0	0	0	0	0	0	0	0	—	—	—	—
*Runx2*	17	SS	LL	SS	LL	578	15	578	15	0	0	0	0	578	15	0	0	0	0	0	0	0	0	—	—	—	—
*Sox5*	6	LL	LL	SS	LL	3,226	115	3,221	111	10	3	1	0	3,175	104	21	4	14	0	5	4	5	0	Np	143,783,835	14	—

Np, no prediction.

a Predicted significance rated by PolyPhen-2, SIFT, and LRT.

b Genotype unassignable.

c Single exon.

## Discussion

This study combines molecular, biological, and genetic approaches to gain insight into genes and physiological pathways involved in the tissue healing, including knee cartilage. The most intriguing finding is that four genes were significantly correlated to both ear wound and articular cartilage healing. These genes represented two important functional classifications: DNA repair (*Pcna*, *Xrcc2*) and Wnt signaling (*Wnt16*, *Axin2*).

The genes involved in DNA repair serve a key function during G2 phase of the cell cycle, including that of chondrocytes (Kim *et al.* 2010). Both *Pcna* and *Xrcc2* play key roles in maintaining chromosome stability, homologous recombination, and cell proliferation at wound sites (Matullo *et al.* 2005). Both *Pcna* and *Xrcc2* have also been implicated in DNA repair deficits in certain cancers (Matullo *et al.* 2005; Moreno *et al.* 2006; Zienolddiny *et al.* 2006). It is plausible that higher expression of these genes in healing strains indicates expedited proliferation of cells (Tchetina 2011), implicating an elevated repair response. Comparing sequence differences between LG/J and SM/J alleles, we noted that *Xrcc2* contains a nonsynonymous change in exon 2 Gly:26:Ala at a highly conserved position, implicating this particular SNP as a functional variant relevant to healing in the LGXSM intercross. In contrast, *Pcna* harbors six SNPs, with only one exonic SNP sitting in a highly conserved site where it produces a synonymous amino acid substitution. Identification of likely functional SNP candidates within *Pcna* will require additional criteria beyond that used here.

The other genes significantly correlated with both phenotypes represented the Wnt signaling pathway. Canonical Wnt signaling is important for embryogenesis, bone metabolism (Baron and Rawadi 2007), and tissue regeneration (Stoick-Cooper *et al.* 2007). Wnt/β-catenin signaling regulates progenitor cell fate and proliferation through embryonic development and stem cell functions (Logan and Nusse 2004). An upregulation of *Wnt16* has been observed in osteoarthritis (Dell’accio *et al.* 2008; Velasco *et al.* 2010) and injured cartilage (Dell’accio *et al.* 2008). *Axin2*, a known inhibitor of the Wnt signaling pathway (Yu *et al.* 2005), suppresses β-catenin and bone remodeling. Knockout of *Axin2* has been shown to result in an age-dependent high bone mass phenotype (Yan *et al.* 2009). In addition, *Axin2* is expressed in developing cartilage and plays an important role in regulating chondrocyte maturation and axial skeletal development (Dao *et al.* 2010). Upregulation of *Axin2* in healers in our study may indicate a differential response of progenitor cells in the bone marrow toward the regenerative process. SNP analysis revealed a SNP occupying a highly conserved position immediately downstream of the *Wnt16* 3′ UTR, and the 5′ UTR contains one highly conserved SNP. Two nonconserved exonic SNPs yield synonymous substitutions in the resulting proteins. The two *Axin2* alleles carried in our RI lines contain 37 SNPs. Out of 30 intronic SNPs, one is highly conserved; however, out of five exonic SNPs, three are highly conserved. A nonsynonymous exonic SNP sits in exon 7 (Arg:648:Cys) and occupies a poorly conserved site. One other exonic SNP produces a nonsynonymous substitution in exon 5 (Tyr:474:His), which is predicted as potentially damaging by the PolyPhen-2 algorithm.

Additionally, we identified four genes correlating with both healing phenotypes, albeit strongly with one yet weakly with the other (Figure 2, Table 1). Expression of *Ulk1* and *Cebpb* correlated significantly positively with cartilage healing, but at a marginal level of significance with ear wound healing. *Il6* and *Map1lc3a* were significantly correlated with ear wound healing in both directions, respectively, yet marginally so with cartilage healing in the positive direction. *Ulk1* is an important regulator of autophagy (Chan *et al.* 2007) and plays a critical role in the autophagic clearance of mitochondria and ribosomes during reticulocyte maturation (Kundu *et al.* 2008). Another important marker and effector of autophagy is *Map1lc3a* (Choi *et al.* 2012). Autophagy genes are repressed in cartilage after aging, injury, osteoarthritis, or mechanical loading (Carames *et al.* 2010, 2012). The upregulation of autophagy genes, especially *Ulk1*, might be associated with increased cell turnover in cartilage and protection from osteoarthritis.

*Il6* is an important player in the development of chronic joint inflammation and is thought to be involved in osteoarthritis-associated joint inflammation (Ohshima *et al.* 1998; de Hooge *et al.* 2000). It has been reported that *Il6* expression can be regulated by *Cebpb* (Natsuka *et al.* 1992). Our laboratory has previously shown that *Cebpb* is associated with cytokine-induced downregulation of extracellular matrix genes in chondrocytes and the repression of cartilage gene expression in noncartilaginous tissues (Okazaki *et al.* 2002, 2006), and is associated with upregulation of chemokines (Zhang *et al.* 2010) and MMP-13 (Okazaki *et al.* 2002). These results indicate a more plausible positive role of *Il6* in wound healing than in cartilage regeneration. Conversely, the role of *Cebpb* appears to be more specific in cartilage repair and osteoarthritis. LG/J and SM/J alleles for both *Il6* and *Cebpb* are identical in their sequences, implying that variations beyond the immediate vicinity, including possible *trans*-polymorphisms, are actually responsible for their variation in expression level among strains. In our RI panel, *Ulk1* harbors four nonconserved SNPs: one synonymous exonic; one intronic; and two within the first 2500 base pairs upstream of the 5′ UTR. *Map1lc3a* harbors 32 SNPs, most of which occur upstream (16 SNPs) and downstream (14 SNPs) of its 5′ UTR and 3′ UTR, respectively. One of the 14 SNPs located downstream of the 3′ UTR is highly conserved. *Map1lc3a* has a single nonconserved intronic SNP but none in exonic regions.

Another nine genes were correlated with both phenotypes at marginal significance (Table 3). Of particular note, *Sox5* harbors an abundance of 3226 SNPs, 115 of which sit in highly conserved sites. The majority of SNPs are intronic. We observed no SNPs between LG/J and SM/J alleles for four genes among those correlating at least marginally with both wound healing phenotypes (*Il6*, *Cebpb*, *Cxcl12*, and *Mmp13*). Strain expression differences with respect to such genes must be attributable to genetic variations outside of the gene proper or *trans*-polymorphism in other genes influencing its expression level, function, or both. Another gene of interest was *p21* (aka *Cdkn1a*), since it has been found that the downregulation of *p21* plays a most striking role in repair as its deletion alone confers regenerative ability (Bedelbaeva *et al.* 2010). In our panel of genes, the expression of *p21* was found to be negatively correlated with both ear wound and articular cartilage repair phenotypes, although it did not reach a statistically significant level. Our findings of its negative correlation with regenerative phenotype and a nonsignificant trend in its expression are in line with the findings of Bedelbaeva. However, there could be two main reasons why our study did not detect a significant relationship between the expression of *p21* and healing potential. First, the activity of *p21* relevant to healing might occur specifically in one of the several tissue types in the knee joint. Because we assayed expression levels from the total knee joint, any tissue-specific effect of *p21* that might have been working toward regeneration could have been diluted out, making it more difficult to detect a statistically significant relationship between overall *p21* expression in the knee joint and healing potential. Second, and more importantly, there is very little genotypic variation in our samples. Although LG/J and SM/J alleles do vary at 54 bases within the *p21* sequence, all the RI lines used in our study are homozygous for the same SM/J allele, except for LGXSM-5, which is heterozygous with one LG/J and one SM/J *p21* allele. Potential functional variation in *p21* is poorly represented in our study, therefore masking its detectable effect on healing.

Another important finding was that the strains can be classified into two main clusters that largely (but not exclusively) correspond to their healing ability (Rai *et al.* 2012). The three top healer strains (except for LGXSM-33) clustered together exclusive of other nonhealers and intermediate healers. This clustering was based on the gene expression variations among RI lines, the basis of which might arise because of functional differences between alleles arising from sequence variations, genetic background, or both.

We have shown that healing responses in both LGXSM-33 and LGXSM-6 closely parallel one another subsequent to injury, but with LGXSM-33 failing to keep pace and halting its progression in the final weeks of healing, during which LGXSM-6 completely regenerates its articular cartilage (Rai *et al.* 2012). Perhaps LGXSM-33 exhibits an expression profile that is quantitatively similar to that of healers, yet it fails to surpass a minimum threshold toward a state attributed with a healing ability.

Our analysis of the broad-sense heritabilities showed that the expressions of one-third of the genes plus all six of the miRNAs were significantly heritable. These results indicate that the gene expression differences observed across the strains are attributable to their genetic differences and are not attributable to environmental, nongenetic factors.

Based on the available genetic map for the LGXSM RI panel (Hrbek *et al.* 2006), we assigned the genotypes for each RI line for each gene correlating (at least marginally) with one or both healing phenotypes. Among the RI lines examined, LGXSM-6 heals the best and LGXSM-33 heals the worst, whereas the other two, LGXSM-5 and LGXSM-35, are intermediate healers. Across genes correlating with healing, we note that LGXSM-6 carries the LG/J allele most often among RI lines. Similarly, the nonhealing LGXSM-33 carries the SM/J allele more often. We surmise that healing ability as a function of the expression of these genes at ear and knee wound sites is conferred mostly by the LG/J allele relative to the SM/J allele.

A limiting aspect of this study was that the tissue lysates for gene expression analyses were prepared from the multiple joint tissues (including cartilage, subchondral bone, meniscus, synovium, joint capsule, and growth plate) instead of just from the articular cartilage. From the tiny mouse knee sections, it was not possible to restrict the analysis only to articular cartilage. Laser capture microdissection (LCM) would have been an alternative strategy, but because we did not have the sections on the LCM-compatible slides, we used whole knee joint tissues, as has been suggested previously (Loeser *et al.* 2012). Although this approach appears to be less sensitive in detecting genes that were differentially regulated exclusively in articular cartilage, and although it might limit the ability to determine which particular tissue contributed to expression of a specific gene, it has the advantage of allowing discovery of genes that are more globally involved in the regenerative process in the knee joint. Another potential limitation of this study is the number of mice (*i.e.*, three to four for each strain) analyzed for gene expression. Because the sample size was not large, we computed the repeatability of the assays. Repeatability is the variance between individuals as a proportion of the variance within individuals (between replicates) plus the variance between individuals. Our repeatability analysis compared the differences among individuals across the whole sample relative to the differences between repeat measures for single individuals (Falconer and Mackay 1996). A higher repeatability value (0.8–0.9) adds confidence to the data for each replicate. Here, we achieved high repeatability values, even to the extent that a single measure would suffice instead of three replicates. Although a few genes had low repeatability values, repeating these measures three times did not provide much improvement (only from 0.63 to 0.73). It is quite possible that these genes were not really expressed at a high enough level to detect differences. Thus, high repeatability of the assay covers the limitation of a low number of mice.

These findings bear great significance for osteoarthritis research. Previously, we have shown that LGXSM-6 (healer) is protected from developing posttraumatic osteoarthritis, whereas the nonhealer (LGXSM-33) strain is susceptible to posttraumatic osteoarthritis (Hashimoto *et al.* 2012). Given the inverse relationship between cartilage healing and osteoarthritis, albeit in a limited set of mouse strains, we suggest that these genes may also be involved in protection from cartilage degeneration as it occurs in osteoarthritis.

In summary, our study has shown that a subset of genes is common to both ear wound and cartilage healing, clustering the variation in gene expression levels classify groups of strains corresponding to healing potential, and the basis of gene expression differences appears to have some genetic component. Given the evidence for common modes of healing in different tissue types, continued genetic and molecular dissection of components of healing will further uncover the mechanism(s) of tissue regeneration.

## Supplementary Material

Supporting Information

## References

[bib1] Adzhubei, I., D. M. Jordan, and S. R. Sunyaev, 2013 Predicting functional effect of human missense mutations using PolyPhen-2. Curr. Protoc. Hum. Genet. Chapter 7: Unit7.20. 10.1002/0471142905.hg0720s76 PMC4480630 23315928

[bib2] Adzhubei I. A. Schmidt S. Peshkin L. Ramensky V. E. Gerasimova A., 2010 A method and server for predicting damaging missense mutations. Nat. Methods 7: 248–249 20354512 10.1038/nmeth0410-248 PMC2855889

[bib3] Baron R. Rawadi G., 2007 Targeting the Wnt/beta-catenin pathway to regulate bone formation in the adult skeleton. Endocrinology 148: 2635–2643 17395698 10.1210/en.2007-0270

[bib4] Bedelbaeva K. Snyder A. Gourevitch D. Clark L. Zhang X. M., 2010 Lack of p21 expression links cell cycle control and appendage regeneration in mice. Proc. Natl. Acad. Sci. USA 107: 5845–5850 20231440 10.1073/pnas.1000830107 PMC2851923

[bib5] Bely A. E., 2010 Evolutionary loss of animal regeneration: pattern and process. Integr. Comp. Biol. 50: 515–527 21558220 10.1093/icb/icq118

[bib6] Blankenhorn E. P. Bryan G. Kossenkov A. V. Clark L. D. Zhang X. M., 2009 Genetic loci that regulate healing and regeneration in LG/J and SM/J mice. Mamm. Genome 20: 720–733 19760323 10.1007/s00335-009-9216-3 PMC3652381

[bib7] Bresters D. Schipper M. E. Reesink H. W. Boeser-Nunnink B. D. Cuypers H. T., 1994 The duration of fixation influences the yield of HCV cDNA-PCR products from formalin-fixed, paraffin-embedded liver tissue. J. Virol. Methods 48: 267–272 7989443 10.1016/0166-0934(94)90125-2

[bib8] Carames B. Hasegawa A. Taniguchi N. Miyaki S. Blanco F. J., 2012 Autophagy activation by rapamycin reduces severity of experimental osteoarthritis. Ann. Rheum. Dis. 71: 575–581 22084394 10.1136/annrheumdis-2011-200557 PMC3294168

[bib9] Carames B. Taniguchi N. Otsuki S. Blanco F. J. Lotz M., 2010 Autophagy is a protective mechanism in normal cartilage, and its aging-related loss is linked with cell death and osteoarthritis. Arthritis Rheum. 62: 791–801 20187128 10.1002/art.27305 PMC2838960

[bib10] Carlson B. M., 2005 Some principles of regeneration in mammalian systems. Anat. Rec. B New Anat. 287: 4–13 16308859 10.1002/ar.b.20079

[bib11] Chan E. Y. Kir S. Tooze S. A., 2007 siRNA screening of the kinome identifies ULK1 as a multidomain modulator of autophagy. J. Biol. Chem. 282: 25464–25474 17595159 10.1074/jbc.M703663200

[bib12] Choi J. Jo M. Lee E. Oh Y. K. Choi D., 2012 The role of autophagy in human endometrium. Biol. Reprod. 86: 70 22088918 10.1095/biolreprod.111.096206

[bib13] Chun S. Fay J. C., 2009 Identification of deleterious mutations within three human genomes. Genome Res. 19: 1553–1561 19602639 10.1101/gr.092619.109 PMC2752137

[bib14] Clark L. D. Clark R. K. Heber-Katz E., 1998 A new murine model for mammalian wound repair and regeneration. Clin. Immunol. Immunopathol. 88: 35–45 9683548 10.1006/clin.1998.4519

[bib15] Colwell A. S. Krummel T. M. Kong W. Longaker M. T. Lorenz H. P., 2006 Skin wounds in the MRL/MPJ mouse heal with scar. Wound Repair Regen. 14: 81–90 16476076 10.1111/j.1524-475X.2005.00092.x

[bib16] Colwell A. S. Longaker M. T. Lorenz H. P., 2005 Mammalian fetal organ regeneration. Adv. Biochem. Eng. Biotechnol. 93: 83–100 15791945 10.1007/b99972

[bib17] Cuervo R. Hernandez-Martinez R. Chimal-Monroy J. Merchant-Larios H. Covarrubias L., 2012 Full regeneration of the tribasal Polypterus fin. Proc. Natl. Acad. Sci. USA 109: 3838–3843 22355122 10.1073/pnas.1006619109 PMC3309738

[bib18] Dao D. Y. Yang X. Flick L. M. Chen D. Hilton M. J., 2010 Axin2 regulates chondrocyte maturation and axial skeletal development. J. Orthop. Res. 28: 89–95 19623616 10.1002/jor.20954 PMC2853598

[bib19] de Hooge A. S. van De Loo F. A. Arntz O. J. van Den Berg W. B., 2000 Involvement of IL-6, apart from its role in immunity, in mediating a chronic response during experimental arthritis. Am. J. Pathol. 157: 2081–2091 11106580 10.1016/S0002-9440(10)64846-8 PMC1885768

[bib20] Dell’accio F. De Bari C. Eltawil N. M. Vanhummelen P. Pitzalis C., 2008 Identification of the molecular response of articular cartilage to injury, by microarray screening: Wnt-16 expression and signaling after injury and in osteoarthritis. Arthritis Rheum. 58: 1410–1421 18438861 10.1002/art.23444

[bib21] Falconer D. S. Mackay T. F. C., 1996 Introduction to Quantitative Genetics, Ed. 4 Longmans Green, Harlow, Essex, UK

[bib22] Fini M. E., 1999 Keratocyte and fibroblast phenotypes in the repairing cornea. Prog. Retin. Eye Res. 18: 529–551 10217482 10.1016/s1350-9462(98)00033-0

[bib23] Finke J. Fritzen R. Ternes P. Lange W. Dolken G., 1993 An improved strategy and a useful housekeeping gene for RNA analysis from formalin-fixed, paraffin-embedded tissues by PCR. Biotechniques 14: 448–453 7681300

[bib24] Fitzgerald J. Rich C. Burkhardt D. Allen J. Herzka A. S., 2008 Evidence for articular cartilage regeneration in MRL/MpJ mice. Osteoarthritis Cartilage 16: 1319–1326 18455447 10.1016/j.joca.2008.03.014

[bib25] Gierer A. Berking S. Bode H. David C. N. Flick K., 1972 Regeneration of hydra from reaggregated cells. Nat. New Biol. 239: 98–101 4507522 10.1038/newbio239098a0

[bib26] Gurtner G. C. Werner S. Barrandon Y. Longaker M. T., 2008 Wound repair and regeneration. Nature 453: 314–321 18480812 10.1038/nature07039

[bib27] Harty M. Neff A. W. King M. W. Mescher A. L., 2003 Regeneration or scarring: an immunologic perspective. Dev. Dyn. 226: 268–279 12557205 10.1002/dvdy.10239

[bib28] Hashimoto S. Rai M. F. Janiszak K. L. Cheverud J. M. Sandell L. J., 2012 Cartilage and bone changes during development of post-traumatic osteoarthritis in selected LGXSM recombinant inbred mice. Osteoarthritis Cartilage 20: 562–571 22361237 10.1016/j.joca.2012.01.022 PMC3353722

[bib29] Heber-Katz E., 1999 The regenerating mouse ear. Semin. Cell Dev. Biol. 10: 415–419 10497098 10.1006/scdb.1999.0328

[bib30] Heydemann A. Swaggart K. A. Kim G. H. Holley-Cuthrell J. Hadhazy M., 2012 The superhealing MRL background improves muscular dystrophy. Skelet Muscle 2: 26 23216833 10.1186/2044-5040-2-26 PMC3534636

[bib31] Hrbek T. de Brito R. A. Wang B. Pletscher L. S. Cheverud J. M., 2006 Genetic characterization of a new set of recombinant inbred lines (LGXSM) formed from the inter-cross of SM/J and LG/J inbred mouse strains. Mamm. Genome 17: 417–429 16688532 10.1007/s00335-005-0038-7

[bib32] Kench J. A. Russell D. M. Fadok V. A. Young S. K. Worthen G. S., 1999 Aberrant wound healing and TGF-beta production in the autoimmune-prone MRL/+ mouse. Clin. Immunol. 92: 300–310 10479535 10.1006/clim.1999.4754

[bib33] Kierdorf U. Kierdorf H., 2011 Deer antlers—a model of mammalian appendage regeneration: an extensive review. Gerontology 57: 53–65 20332600 10.1159/000300565

[bib34] Kim J. Xu M. Xo R. Mates A. Wilson G. L., 2010 Mitochondrial DNA damage is involved in apoptosis caused by pro-inflammatory cytokines in human OA chondrocytes. Osteoarthritis Cartilage 18: 424–432 19822235 10.1016/j.joca.2009.09.008

[bib35] Knudsen B. S. Allen A. N. McLerran D. F. Vessella R. L. Karademos J., 2008 Evaluation of the branched-chain DNA assay for measurement of RNA in formalin-fixed tissues. J. Mol. Diagn. 10: 169–176 18276773 10.2353/jmoldx.2008.070127 PMC2259472

[bib36] Kumar P. Henikoff S. Ng P. C., 2009 Predicting the effects of coding non-synonymous variants on protein function using the SIFT algorithm. Nat. Protoc. 4: 1073–1081 19561590 10.1038/nprot.2009.86

[bib37] Kundu M. Lindsten T. Yang C. Y. Wu J. Zhao F., 2008 Ulk1 plays a critical role in the autophagic clearance of mitochondria and ribosomes during reticulocyte maturation. Blood 112: 1493–1502 18539900 10.1182/blood-2008-02-137398 PMC2515143

[bib38] Li X. Gu W. Masinde G. Hamilton-Ulland M. Xu S., 2001 Genetic control of the rate of wound healing in mice. Heredity (Edinb) 86: 668–674 11595047 10.1046/j.1365-2540.2001.00879.x

[bib39] Loeser R. F. Olex A. L. McNulty M. A. Carlson C. S. Callahan M. F., 2012 Microarray analysis reveals age-related differences in gene expression during the development of osteoarthritis in mice. Arthritis Rheum. 64: 705–717 21972019 10.1002/art.33388 PMC3269534

[bib40] Logan C. Y. Nusse R., 2004 The Wnt signaling pathway in development and disease. Annu. Rev. Cell Dev. Biol. 20: 781–810 15473860 10.1146/annurev.cellbio.20.010403.113126

[bib41] Macabeo-Ong M. Ginzinger D. G. Dekker N. McMillan A. Regezi J. A., 2002 Effect of duration of fixation on quantitative reverse transcription polymerase chain reaction analyses. Mod. Pathol. 15: 979–987 12218216 10.1097/01.MP.0000026054.62220.FC

[bib42] Masinde G. L. Li R. Nguyen B. Yu H. Srivastava A. K., 2006 New quantitative trait loci that regulate wound healing in an intercross progeny from DBA/1J and 129 x 1/SvJ inbred strains of mice. Funct. Integr. Genomics 6: 157–163 16208538 10.1007/s10142-005-0004-1

[bib43] Masuda N. Ohnishi T. Kawamoto S. Monden M. Okubo K., 1999 Analysis of chemical modification of RNA from formalin-fixed samples and optimization of molecular biology applications for such samples. Nucleic Acids Res. 27: 4436–4443 10536153 10.1093/nar/27.22.4436 PMC148727

[bib44] Matullo G. Guarrera S. Sacerdote C. Polidoro S. Davico L., 2005 Polymorphisms/haplotypes in DNA repair genes and smoking: a bladder cancer case-control study. Cancer Epidemiol. Biomarkers Prev. 14: 2569–2578 16284380 10.1158/1055-9965.EPI-05-0189

[bib45] McBrearty B. A. Clark L. D. Zhang X. M. Blankenhorn E. P. Heber-Katz E., 1998 Genetic analysis of a mammalian wound-healing trait. Proc. Natl. Acad. Sci. USA 95: 11792–11797 9751744 10.1073/pnas.95.20.11792 PMC21719

[bib46] Metcalfe A. D. Willis H. Beare A. Ferguson M. W., 2006 Characterizing regeneration in the vertebrate ear. J. Anat. 209: 439–446 17005017 10.1111/j.1469-7580.2006.00632.x PMC2100363

[bib47] Moreno V. Gemignani F. Landi S. Gioia-Patricola L. Chabrier A., 2006 Polymorphisms in genes of nucleotide and base excision repair: risk and prognosis of colorectal cancer. Clin. Cancer Res. 12: 2101–2108 16609022 10.1158/1078-0432.CCR-05-1363

[bib48] Murphy E. D. Roths J. B., 1979 Autoimmunity and lymphoproliferation: Induction by mutant gene lpr and acceleration by a male-associated factor in strain BXSB, pp. 207–220 in Genetic Control of Autoimmune Disease, edited by Rose N. R. Bigazzi P. E. Warner N. L. Elsevier, New York

[bib49] Natsuka S. Akira S. Nishio Y. Hashimoto S. Sugita T., 1992 Macrophage differentiation-specific expression of NF-IL6, a transcription factor for interleukin-6. Blood 79: 460–466 1730090

[bib50] Ohshima S. Saeki Y. Mima T. Sasai M. Nishioka K., 1998 Interleukin 6 plays a key role in the development of antigen-induced arthritis. Proc. Natl. Acad. Sci. USA 95: 8222–8226 9653168 10.1073/pnas.95.14.8222 PMC20957

[bib51] Okazaki K. Li J. Yu H. Fukui N. Sandell L. J., 2002 CCAAT/enhancer-binding proteins beta and delta mediate the repression of gene transcription of cartilage-derived retinoic acid-sensitive protein induced by interleukin-1 beta. J. Biol. Chem. 277: 31526–31533 12072435 10.1074/jbc.M202815200

[bib52] Okazaki K. Yu H. Davies S. R. Imamura T. Sandell L. J., 2006 A promoter element of the CD-RAP gene is required for repression of gene expression in non-cartilage tissues in vitro and in vivo. J. Cell. Biochem. 97: 857–868 16250001 10.1002/jcb.20648

[bib53] Park Y. N. Abe K. Li H. Hsuih T. Thung S. N., 1996 Detection of hepatitis C virus RNA using ligation-dependent polymerase chain reaction in formalin-fixed, paraffin-embedded liver tissues. Am. J. Pathol. 149: 1485–1491 8909238 PMC1865282

[bib54] Rai M. F. Hashimoto S. Johnson E. E. Janiszak K. L. Fitzgerald J., 2012 Heritability of articular cartilage regeneration and its association with ear wound healing in mice. Arthritis Rheum. 64: 2300–2310 22275233 10.1002/art.34396 PMC3360138

[bib55] Rai M. F. Patra D. Sandell L. J. Brophy R. H., 2013 Transcriptome analysis of injured human meniscus reveals a distinct phenotype of meniscus degeneration with aging. Arthritis Rheum. 65: 2090–2101 23658108 10.1002/art.37984 PMC3873730

[bib56] Sanchez Alvarado A. Tsonis P. A., 2006 Bridging the regeneration gap: genetic insights from diverse animal models. Nat. Rev. Genet. 7: 873–884 17047686 10.1038/nrg1923

[bib57] Seifert A. W. Monaghan J. R. Smith M. D. Pasch B. Stier A. C., 2012 The influence of fundamental traits on mechanisms controlling appendage regeneration. Biol. Rev. Camb. Philos. Soc. 87: 330–345 21929739 10.1111/j.1469-185X.2011.00199.x

[bib58] Stocum D. L., 1984 The urodele limb regeneration blastema. Determination and organization of the morphogenetic field. Differentiation 27: 13–28 6381198 10.1111/j.1432-0436.1984.tb01403.x

[bib59] Stoick-Cooper C. L. Weidinger G. Riehle K. J. Hubbert C. Major M. B., 2007 Distinct Wnt signaling pathways have opposing roles in appendage regeneration. Development 134: 479–489 17185322 10.1242/dev.001123

[bib60] Tanaka E. M., 2003 Regeneration: if they can do it, why can’t we? Cell 113: 559–562 12787496 10.1016/s0092-8674(03)00395-7

[bib61] Tchetina E. V., 2011 Developmental mechanisms in articular cartilage degradation in osteoarthritis. Arthritis (Egypt) 2011: 683970 10.1155/2011/683970 PMC3199933 22046522

[bib62] ten Koppel P. G. van Osch G. J. Verwoerd C. D. Verwoerd-Verhoef H. L., 2001 A new in vivo model for testing cartilage grafts and biomaterials: the ’rabbit pinna punch-hole’ model. Biomaterials 22: 1407–1414 11336315 10.1016/s0142-9612(00)00298-2

[bib63] Urdea M. S. Horn T. Fultz T. J. Anderson M. Running J. A., 1991 Branched DNA amplification multimers for the sensitive, direct detection of human hepatitis viruses. Nucleic Acids Symp. Ser. 24: 197–200 1668687

[bib64] Velasco J. Zarrabeitia M. T. Prieto J. R. Perez-Castrillon J. L. Perez-Aguilar M. D., 2010 Wnt pathway genes in osteoporosis and osteoarthritis: differential expression and genetic association study. Osteoporos. Int. 21: 109–118 19373426 10.1007/s00198-009-0931-0

[bib65] Ward B. D. Furman B. D. Huebner J. L. Kraus V. B. Guilak F., 2008 Absence of posttraumatic arthritis following intraarticular fracture in the MRL/MpJ mouse. Arthritis Rheum. 58: 744–753 18311808 10.1002/art.23288

[bib66] Yan Y. Tang D. Chen M. Huang J. Xie R., 2009 Axin2 controls bone remodeling through the beta-catenin-BMP signaling pathway in adult mice. J. Cell Sci. 122: 3566–3578 19737815 10.1242/jcs.051904 PMC2746134

[bib67] Yang W. Maqsodi B. Ma Y. Bui S. Crawford K. L., 2006 Direct quantification of gene expression in homogenates of formalin-fixed, paraffin-embedded tissues. Biotechniques 40: 481–486 16629395 10.2144/000112133

[bib68] Yu H. M. Jerchow B. Sheu T. J. Liu B. Costantini F., 2005 The role of Axin2 in calvarial morphogenesis and craniosynostosis. Development 132: 1995–2005 15790973 10.1242/dev.01786 PMC1828115

[bib69] Zhang Z. Bryan J. L. DeLassus E. Chang L. W. Liao W., 2010 CCAAT/Enhancer-binding protein {beta} and NF-{kappa}B mediate high level expression of chemokine genes CCL3 and CCL4 by human chondrocytes in response to IL-1{beta}. J. Biol. Chem. 285: 33092–33103 20702408 10.1074/jbc.M110.130377 PMC2963416

[bib70] Zienolddiny S. Campa D. Lind H. Ryberg D. Skaug V., 2006 Polymorphisms of DNA repair genes and risk of non-small cell lung cancer. Carcinogenesis 27: 560–567 16195237 10.1093/carcin/bgi232

