# Supplementary material for: Molecular Insight into the Association Between Cartilage Regeneration and Ear Wound Healing in Genetic Mouse Models: Targeting New Genes in Regeneration
Source: G3 (Bethesda). 2013 Nov 1;3(11):1881–91. doi: 10.1534/g3.113.007302 (PMC3815053; doi:10.1534/g3.113.007302)
Supplement: Supporting Information [file supp_3_11_1881__index.html]

Molecular Insight into the Association Between Cartilage Regeneration and Ear Wound Healing in Genetic Mouse Models: Targeting New Genes in Regeneration — Supporting Information 

# Molecular Insight into the Association Between Cartilage Regeneration and Ear Wound Healing in Genetic Mouse Models: Targeting New Genes in Regeneration

## Supporting Information for Rai *et al.*, 2013

**Files in this Data Supplement:**

- Table S1 - Ear Wound Phenotype (.xls, 56 KB)
- Table S2 - Articular Cartilage Phenotype (.xls, 75 KB)
- Table S3 - Characteristics of mRNA probes (.xls, 41 KB)
- Table S4 - Characteristics of miRNA probes (.xls, 30 KB)
